# Lestaurtinib Inhibition of the JAK/STAT Signaling Pathway in Hodgkin Lymphoma Inhibits Proliferation and Induces Apoptosis

**DOI:** 10.1371/journal.pone.0018856

**Published:** 2011-04-20

**Authors:** Tania Diaz, Alfons Navarro, Gerardo Ferrer, Bernat Gel, Anna Gaya, Rosa Artells, Beatriz Bellosillo, Mar Garcia-Garcia, Sergi Serrano, Antonio Martínez, Mariano Monzo

**Affiliations:** 1 Human Anatomy Unit, Molecular Oncology and Embryology Laboratory, University of Barcelona Medical School, IDIBAPS, Barcelona, Spain; 2 Institute of Hematology and Oncology, Hospital Clinic, IDIBAPS, Barcelona, Spain; 3 Software Department, Universitat Politecnica de Catalunya, Barcelona, Spain; 4 Pathology Department, Hospital del Mar, Barcelona, Spain; 5 Hematopathology Section, Laboratory of Pathology, Hospital Clinic, IDIBAPS, Barcelona, Spain; University Hospital Vall d'Hebron, Spain

## Abstract

Standard cytotoxic chemotherapy for Hodgkin Lymphoma (HL) has changed little in 30 years; the treatment for patients with relapsed or refractory disease remains challenging and novel agents are under development. JAK/STAT constitutive activation plays an important role in the pathogenesis of HL. Lestaurtinib is an orally bioavailable multikinase inhibitor that has recently been shown to inhibit JAK2 in myeloproliferative disorders. The potential role of Lestaurtinib in HL therapy is unknown. We have analyzed the effect of Lestaurtinib treatment in five HL cell lines from refractory patients, L-428, L-1236, L-540, HDML-2 and HD-MY-Z. At 48 h, a dose-dependent cell growth inhibition (23%–66% at 300 nM) and apoptotic increment (10%–64% at 300 nM) were observed. Moreover, Lestaurtinib inhibited JAK2, STAT5 and STAT3 phosphorylation and reduced the mRNA expression of its downstream antiapoptotic target Bcl-xL. In addition, we have analyzed the effect of Lestaurtinib treatment in lymph nodes from four classic HL patients. We observed a decrease in cell viability at 24 hours of treatment in three patients (mean decrease of 27% at 300 nM). Our findings provide, for the first time, a molecular rationale for testing JAK2 inhibitors, specifically Lestaurtinib, in HL patients.

## Introduction

Hodgkin lymphoma (HL) is characterized by the presence of a small proportion of tumor cells, the Hodgkin/Reed Sternberg (HRS) cells, surrounded by a specific non-tumor microenvironment. HRS cells usually account for only 1% of cells in the tumor tissue, and few cell lines have been established from HL patients. To date, the most frequent genetic alterations in HRS cells involve members of two main signaling pathways: nuclear factor-kappaB (NF-κB) and Janus kinase-Signal transducer and activator of transcription (JAK/STAT) [1].

The JAK2/STAT5 pathway is a common signaling pathway used by many cytokines that regulate target gene expression related to cell survival, proliferation, angiogenesis, and immune evasion [2]. Bcl-xL is an antiapoptotic gene whose expression is induced by STAT5 DNA binding, and activation of the JAK2/STAT5 pathway can modulate apoptosis and survival through Bcl-xL expression [3]. The JAK2/STAT5 pathway plays an active role in HL, where genomic gains of JAK2 are frequently observed [4], and where the suppressor of cytokine signaling 1 (SOCS1), a negative regulator of JAK/STAT signaling, appears mutated and inactivated [5]. While activating mutations in JAK2 have been found in myeloproliferative disorders (MPD) [6], the expression of JAK2 in primary mediastinal large B-cell lymphomas and HL is not the result of mutations [7], although constitutive activation of STATs has been observed [8]. Recently, our group has observed a postranscriptional regulation of JAK2 mediated by a microRNA (miRNA), miR-135a, whose expression was downregulated in HL patients [9].

Although HL is considered one of the most curable human cancers (cure rates of 80–90%) [10], the treatment of patients with relapsed and refractory disease, especially those who relapse after autologous stem cell transplantation, remains challenging. The gold-standard therapy in HL is anthracycline-based, with doxorubicin, bleomycin, vinblastine and dacarbacine (ABVD) [11]. HL patients whose disease relapses after stem cell transplantation are rarely cured with current treatment modalities. Moreover, no new drugs have been approved for HL by the US Food and Drug Administration (FDA) in more than 30 years [12]. Thus, new drugs and novel treatment strategies based on our understanding of HL biology and signaling pathways are needed to improve outcome for these patients. Several therapeutic targets, including JAK2, have been identified and continue to be studied [4], [13]. Novel JAK2 inhibitors have been developed and tested [14], some of which are now being studied in phase I clinical trials in HL [15].

Lestaurtinib (formerly known as CEP-701) is a multi-targeted tyrosine kinase inhibitor which has been shown to potently inhibit FLT3 at nanomolar concentrations in preclinical studies, leading to its rapid development as a potential targeted agent in acute myeloid leukemia [16]. Moreover, recent studies have further shown that Lestaurtinib inhibitory activity is not limited to FLT3 and can suppress JAK2/STAT5 signaling through specific JAK2 inhibition [17]. In order to elucidate the potential role of Lestaurtinib in HL, we have analyzed the *in vitro* effectiveness of Lestaurtinib in five HL cell lines from refractory patients and its role in the JAK2/STAT5 signaling pathway. In addition, we have analyzed for the first time the effect of Lestaurtinib in lymph nodes from classic HL patients by flow cytometry.

## Materials and Methods

### Cell culture and treatment

Five HL cell lines, L-428, L-1236, L-540, HDLM-2 and HD-MY-Z (DSMZ - the German Resource Centre for Biological Material) were assayed for proliferation and apoptosis after treatment with Lestaurtinib (CEP-701 hydrate, Sigma-Aldrich, St. Louis, MO) or DMSO (Sigma-Aldrich). L-428, L-1236 and HDMYZ cell lines, were cultured in RPMI 1640 containing 10% fetal calf serum (Invitrogen, Paisley, UK); the L-540 and HDLM2 cell lines were cultured in RPMI 1640 containing 20% fetal calf serum (Invitrogen). For proliferation and apoptosis analyses, cells (1×10^5^ cells/well) were plated in a 96-well plate in culture medium in the presence of 30, 50, 70, 100, 150, 200 or 300 nM Lestaurtinib or no drug/DMSO vehicle control. In addition, in order to compare the effect of Lestaurtinib with doxorubicin, a component of standard HL therapy, we performed a proliferation analysis in cells treated with 300 nM of doxorubicin. For protein analysis, cells (1.5×10^6^ cells/well) were plated in a 12-well plate and treated with 30, 100 or 300 nM Lestaurtinib or 300 nM DMSO. All cells were treated with a unique dose at the start of the experiment, after which the medium was not modified or replaced.

### Proliferation and apoptosis assays

Cell growth was determined by the CellTiter 96 AQueous One Solution Cell Proliferation Assay (MTS) (Promega, Madison, WI). At 48 h after treatment with Lestaurtinib or DMSO, MTS reagent was added and cells were incubated for 30–60 mins at 37°C. Cell proliferation was measured by OD 490 nm using a VersaMax microplate reader (Molecular Devices, Silicon Valley, CA).

Caspase 3/7 activity was directly measured at 48 h after treatment using a CaspaseGlo 3/7 kit (Promega) as per the manufacturer's protocol. At 48 h after treatment with Lestaurtinib or DMSO, CaspaseGlo reagent was added and cells were incubated for 1 hour at room temperature in the dark. Relative light intensity was measured in each well using an Orion II Microplate luminometer (Berthold Detection Systems, Black Forest, Germany).

### Western Blot analysis

Total protein was isolated using Qiagen Qproteome Mammalian Protein Prep Kit (Qiagen, Hilden, Germany) according to the manufacturer's protocol. Equal amounts of proteins (50 µg) were separated by SDS-polyacrylamide electrophoresis in 10% Tris-HCl polyacrylamide gels and transferred to pure nitrocellulose membranes (Trans-Blot Transfer Medium, Bio-Rad, Hercules, CA). Membranes were incubated with antibodies against JAK2 (Upstate, Millipore, Billerica, MA), phospho-JAK2 (Tyr1007/1008), phospho-STAT5 (Tyr694) and phospho-STAT3 (Tyr705) (Cell Signaling, Danvers, MA), STAT5 and STAT3 (Santa Cruz Biotechonology, Santa Cruz, CA) and á-tubulin (Sigma) as control. Antibody binding was revealed by incubation with anti-mouse (Sigma) or anti-rabbit (Santa Cruz Biotechnology) IgG peroxidase conjugate secondary antibodies. Chemiluminescence was detected using SuperSignal West Pico Chemiluminescent Substrate (Pierce Biotechnology, Rockford, IL) and read in Chemidoc System (Bio-Rad). The protein density of the bands was quantified using Quantity One software v 4.2.6, and relative quantification was calculated with reference to the á-tubulin signal.

### Bcl-xL mRNA analysis

RNA was extracted from the cell lines using Trizol total RNA isolation reagent (Invitrogen, Carlsbad, CA) as per the manufacturer's protocol. Total cDNA was synthesized from total RNA using the High Capacity cDNA Reverse Transcription Kit (Applied Biosystems, Foster City, CA) as per the manufacturer's protocol. Polymerase chain reaction (PCR) was performed using TaqMan Gene Expression assays (Applied Biosystems) for BCL-xL (Hs99999146_m1) and GUSB (Hs99999908_m1), used as endogenous control.

### Patient lymph node analysis

A cell suspension was made from cryopreserved (freezing medium based on RPMI 30%, FBS 60% and DMSO 10%), newly diagnosed lymph node samples from four classic HL patients diagnosed at the Hematology Department of the University Hospital del Mar, Barcelona, Spain (Table 1). The study was approved by the local Ethics Committee and informed consent was provided according to the Declaration of Helsinki.

**Table 1:** Clinical and biological characteristics of HL patients.

Characteristic	P1	P2	P3	P4
**Age**	24	30	28	43
**Sex**	Female	Male	Male	Female
**Histology**	Classic HL, Nodular sclerosis	Classic HL*	Classic HL, Lymphocyte-rich	Classic HL, Nodular sclerosis
**EBV**	−	−	−	−
**CD30**	+	+	+	+
**CD15**	+	+	+	+
**CD20**	−	−	−	−
**Stage**	IIA	IIIA	IIIA	IIA

*Unknown histologic subtype.

The viability of the lymph node cells was at least 87% at the initiation of cell culture, as assessed by Tripan Blue (Invitrogen). We performed a cell viability analysis of lymph node cells and found that viability was 59.9% at baseline, 41.8% at 24 h, and 30.6% at 48 h (Figure S3). Based on these results, we evaluated by flow cytometry 750,000 cells cultured for 24 hours with 300 nM of Lestaurtinib or DMSO. HRS cells were gated by the expression of CD40-PE-Cy5, CD95-Pacific Blue and CD30-PE, and the absence of CD3-APC-Cy7. Antibodies and control isotypes were obtained from BD Bioscience (Franklin Lakes, NJ) and Biolegend (San Diego, CA) [18], [19]. Viability was analyzed by the presence of the membrane phospholipid phosphatidylserine on the outer leaflet of the plasma membrane, using FITC Annexin V (BD Bioscience). In order to obtain an accurate count, we added the same number of CountBright™ absolute counting beads (Invitrogen) to each sample. Samples were analyzed on a FACS CANTO II (Becton Dickinson) and 200,000 events were collected.

### Statistical analysis

Means were compared between 2 groups using a 2-sided Student t-test, using GraphPad Prism 5 (GraphPad Software, Inc., La Jolla, CA). The proliferation and apoptosis data were shown as mean ± SEM of three independent replicates.

## Results and Discussion

Although HL is a highly curable disease, advanced HL has typically been associated with high failure rates [20] and relapsed or refractory HL constitutes a common problem [21], [22]; new drugs are thus needed for these groups of patients. In the present study, we analyzed the *in vitro* activity of Lestaurtinib, which has recently been shown to be a multikinase inhibitor that targets both wild-type and mutated JAK2 in MPD [16].

**Figure 1:**
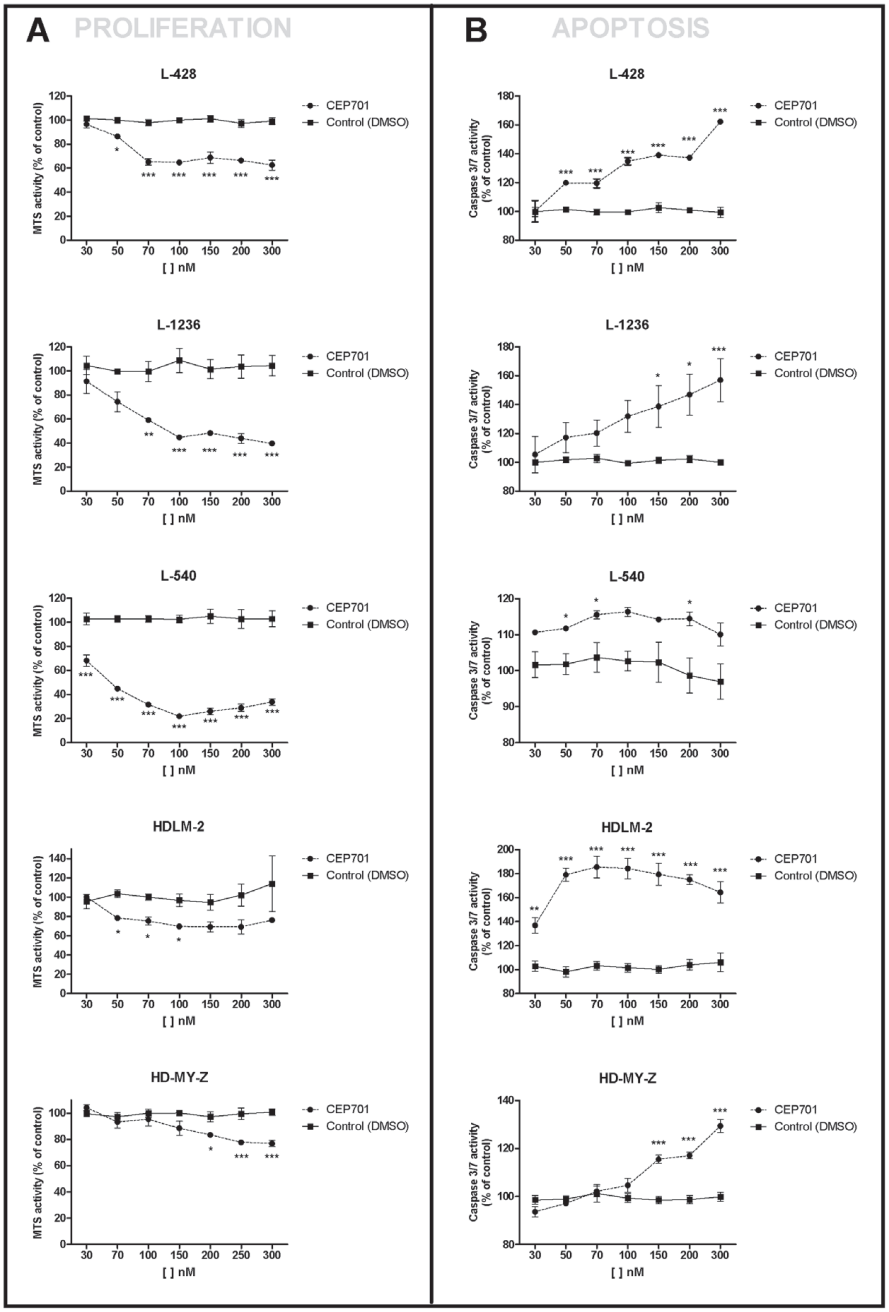
Proliferation (A) and apoptosis (B) analysis after 48 h of Lestaurtinib treatment in L-428, L-1236, L-540, HDLM-2 and HD-MY-Z cell lines. The data are shown as mean ± SEM of three independent replicates. *p<0.05; **p<0.01; ***p<0.001.

Proliferation and apoptosis in response to Lestaurtinib of cultured HL cells was evaluated in all HL cell lines after 48 h of treatment and compared to cells treated with DMSO vehicle control (normalized to 100%). Reduction of proliferation of HL cells reached the lowest level at 100 nM of Lestaurtinib and remained constant thereafter (Figure 1A). At 300 nM of Lestaurtinib, a 38% reduction in proliferation was observed in L-428, 60% in L-1236, 66% in L-540, 23% in HDLM-2 and 23% in HD-MY-Z cell lines. At 300 nM of doxorubicin, the reduction in proliferation was 20.3% in L-428, 18.7% in L-1236, 54% in L-540, 34.5% in HDLM-2, and 19% in HD-MY-Z cell lines. At 300 nM, apoptosis increased 62% in L-428, 57% in L-1236, 10% in L-540, 64% in HDLM-2 and 30% in HD-MY-Z (Figure 1B). In order to determine whether Lestaurtinib inhibition had a transient or a long-lasting effect on cell growth, we then looked at the effect at different incubation times (24, 48, 72 and 96 h). We observed no significant differences between 48, 72 or 96 hours (Figure S1).

Proliferation and apoptosis were both Lestaurtinib dose-dependent. Since DMSO is toxic at doses of 5 µM, it was impossible to determine the dose at which 100% of the cells were killed. However, 50% of the cells had died at 1 µM in L-428 and at 300 nM in L-1236 and L-540, while, in HDLM-2 and HD-MY-Z, about 45% and 42% of cells, respectively, had died at 4 µM (Figure S2).

**Figure 2:**
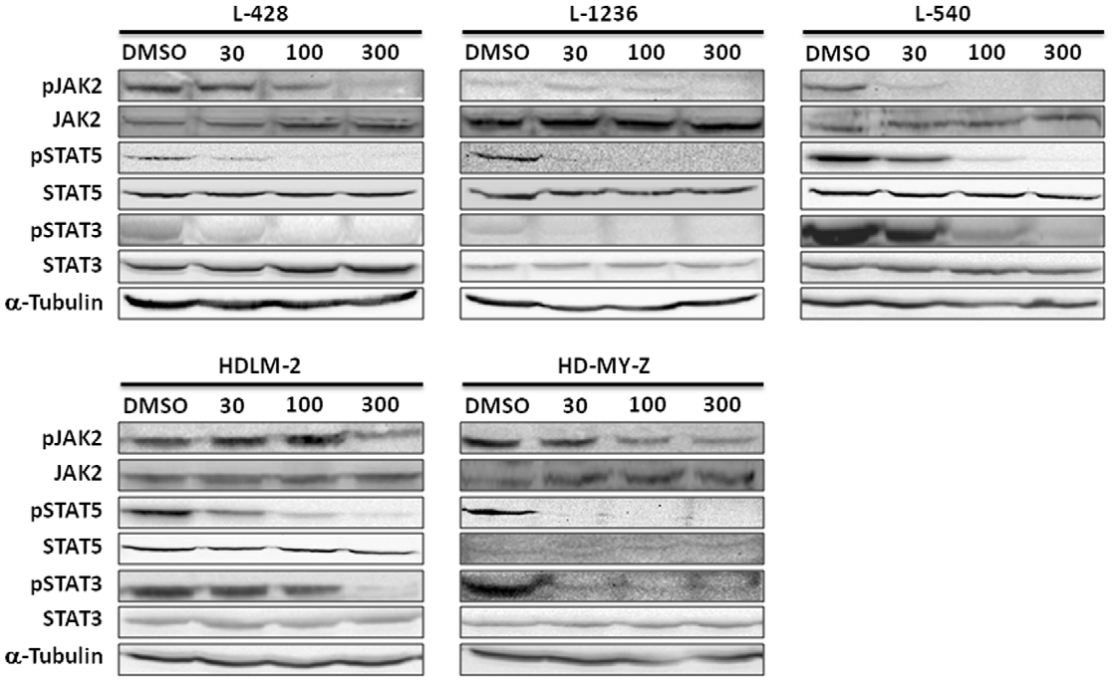
Western blot analysis of JAK2/STAT5 pathway protein levels in L-428, L-1236, L-540, HDLM-2 and HD-MY-Z cells after 1 h of Lestaurtinib treatment at different doses: 30, 100 and 300 nM.

The JAK/STAT pathway is one of the most frequently altered pathways in HL. In addition to genomic gains of JAK2 [4], particularly due to 9p24 gains [23], SOCS1, a negative regulator of JAK/STAT signaling, is often somatically mutated and inactivated [5]. Moreover, constitutive activation of STAT3 has been reported in HL cell lines [8]. In order to investigate the effect of Lestaurtinib treatment on the JAK2 pathway, we assessed the levels and the phosphorylation state of JAK2 and its downstream target molecules following Lestaurtinib treatment. After 1 h, phospho-JAK2 levels had decreased in all the HL cell lines by 46–94% at 300 nM, although no significant changes were observed in JAK2 total protein expression (Figure 2). To assess in greater detail the effects of Lestaurtinib-mediated JAK2 inhibition on the JAK2/STAT5 signaling pathway, protein levels of STAT5, phospho-STAT5, STAT3 and phospho-STAT3 were then analyzed (Figure 2). Lestaurtinib significantly inhibited phosphorylation of STAT5 and STAT3, but with no significant changes in STAT5 and STAT3 total protein. Following 1 hour of 300 nM of Lestaurtinib treatment, phospho-STAT5 and phospho-STAT3 levels decreased by 88–100% and by 97–100%, respectively (Figure 2).

**Figure 3:**
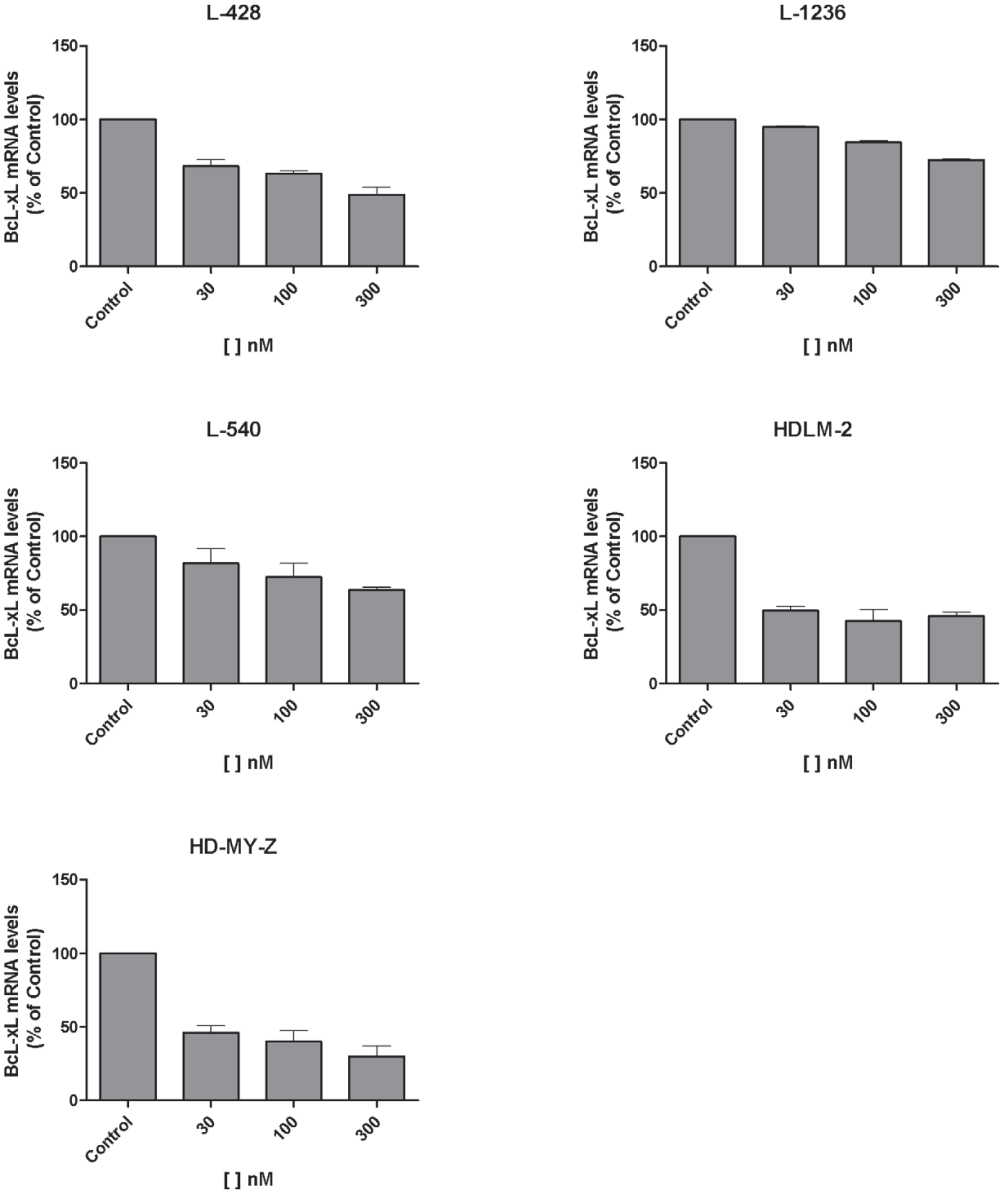
Bcl-xL mRNA analysis after 1 h of Lestaurtinib treatment in L-428, L-1236, L-540, HDLM-2 and HD-MY-Z cell lines.

Bcl-xL is a prosurvival protein induced by phosphorylated STAT5 DNA binding. It appears upregulated in some hematologic malignancies [24] and in primary HL samples [25] and is involved in apoptotic resistance in HRS cells [25]. Decreased phosphorylation of STAT5 resulted in decreased mRNA expression of its downstream antiapoptotic effector Bcl-xL. After 1 h of 300 nM of Lestaurtinib treatment, Bcl-xL mRNA expression levels had decreased by 52% in L-428, 28% in L-1236, 37% in L-540, 55% in HDLM-2 and 71% in HD-MY-Z (Figure 3). This downregulation of Bcl-xL could explain the proapoptotic effect of Lestaurtinib [26].

**Figure 4:**
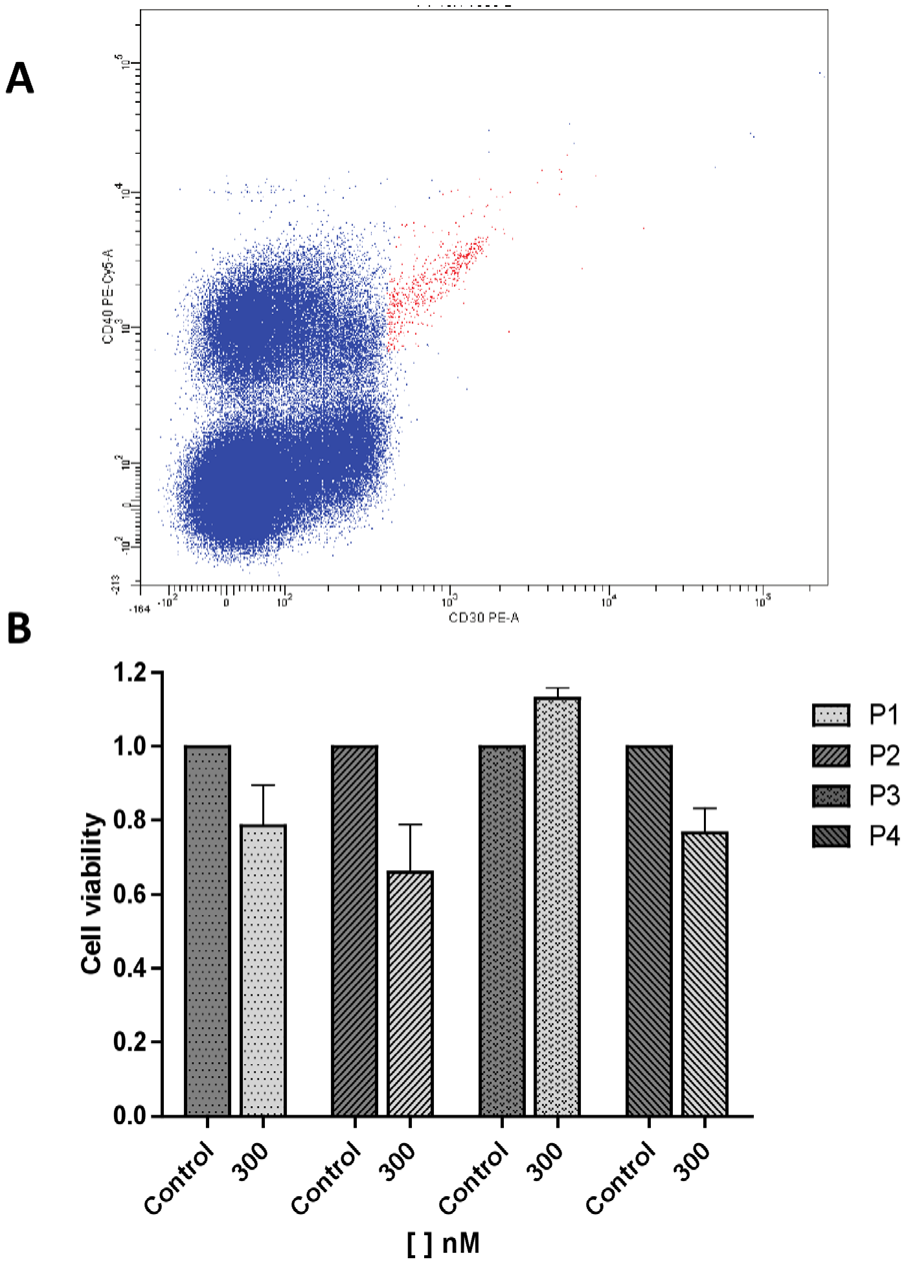
Analysis of cell viability after 24 h of Lestaurtinib treatment in four lymph nodes from classic HL patients. A: representative example of population selected for analysis (anexin-negative, CD3−, CD40+, CD30+ and CD95+). B: cell viability after treatment compared to DMSO control. The data are shown as mean ± SEM of two independent replicates. SEM was calculated on the proportion (treated/untreated cells).

Additionally, we have analyzed the effect of Lestaurtinib in lymph nodes from four classic HL patients. Fromm et al. demonstrated that HL cells from a lymph node can be detected [19] and sorted by flow cytometry [18]. In the present study, we have evaluated the effect of treatment with 300 nM of Lestaurtinib in the subpopulation of lymph node cells CD30+, CD40+, CD95+ and CD3-, which contain HL cells [18], [19] (Figure 4A).

After 24 h, cell viability had decreased in three of the four cases by 22%, 35% and 24% versus control cells (DMSO) (Figure 4B). In the patient 3 (non-responder), we increased the treatment dose to 1 µM and then we observed a reduction in cell viability by 12% (SEM ± 2,3%). This patient has a different histological subtype (lymphocyte-rich) than cell lines (nodular sclerosis and mixed cellularity), and this could explain the different treatment response. In order to shed light on the potential toxicity of Lestaurtinib, we have also analyzed cell viability in lymph node CD3+ cells after treatment with 300 nM of Lestaurtinib and observed no decrease of viability (mean versus control = 100.5%; range: 90%–119%).

The present study is the first of its kind to analyze treatment of HL in patient lymph nodes by flow cytometry. Although our results cannot be conclusive due to our small sample size, they provide the first hints that Lestaurtinib induces growth inhibition and apoptosis activation in HL cells through dysregulation of the JAK2/STAT5 signaling pathway. If our findings are confirmed in a larger patient population, they could provide a molecular rationale for considering treatment with Lestaurtinib for HL patients with relapsed/refractory disease.

## Supporting Information

Figure S1 Proliferation analysis after 24 h, 48 h, 72 h and 96 h of Lestaurtinib treatment in L-428, L-1236, L-540, HDLM-2 and HD-MY-Z cell lines. The data are shown as mean ± SEM of three independent replicates.
(TIF)

Figure S2 Proliferation analysis after 48 h of Lestaurtinib treatment at increasing doses up to 4 µM in L-428, L-1236, L-540, HDLM-2 and HD-MY-Z cell lines. The data are shown as mean ± SEM of three independent replicates.
(TIF)

Figure S3 Cell viability analysis (negative Annexin V) of lymph node cells cultured up to 48 h with growth media (RPMI1640 with 10% FBS).
(TIF)
