# Supplementary figures and images for: Lestaurtinib Inhibition of the JAK/STAT Signaling Pathway in Hodgkin Lymphoma Inhibits Proliferation and Induces Apoptosis
Source: PLoS One. 2011 Apr 20;6(4):e18856. doi: 10.1371/journal.pone.0018856 (PMC3080386; doi:10.1371/journal.pone.0018856)

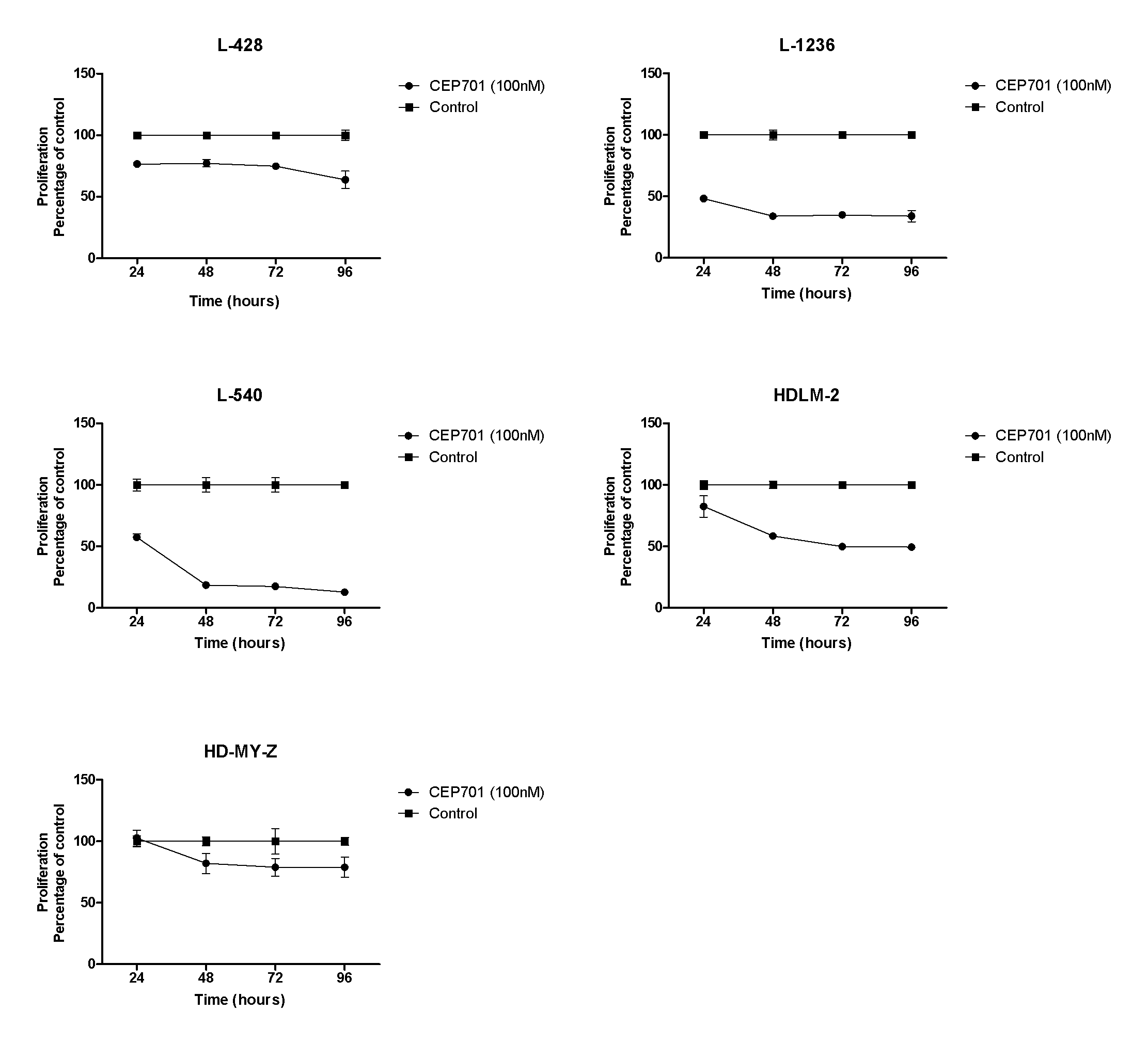

Supplement: Figure S1 — Proliferation analysis after 24 h, 48 h, 72 h and 96 h of Lestaurtinib treatment in L-428, L-1236, L-540, HDLM-2 and HD-MY-Z cell lines. The data are shown as mean ± SEM of three independent replicates. (TIF) [file pone.0018856.s001.tif]

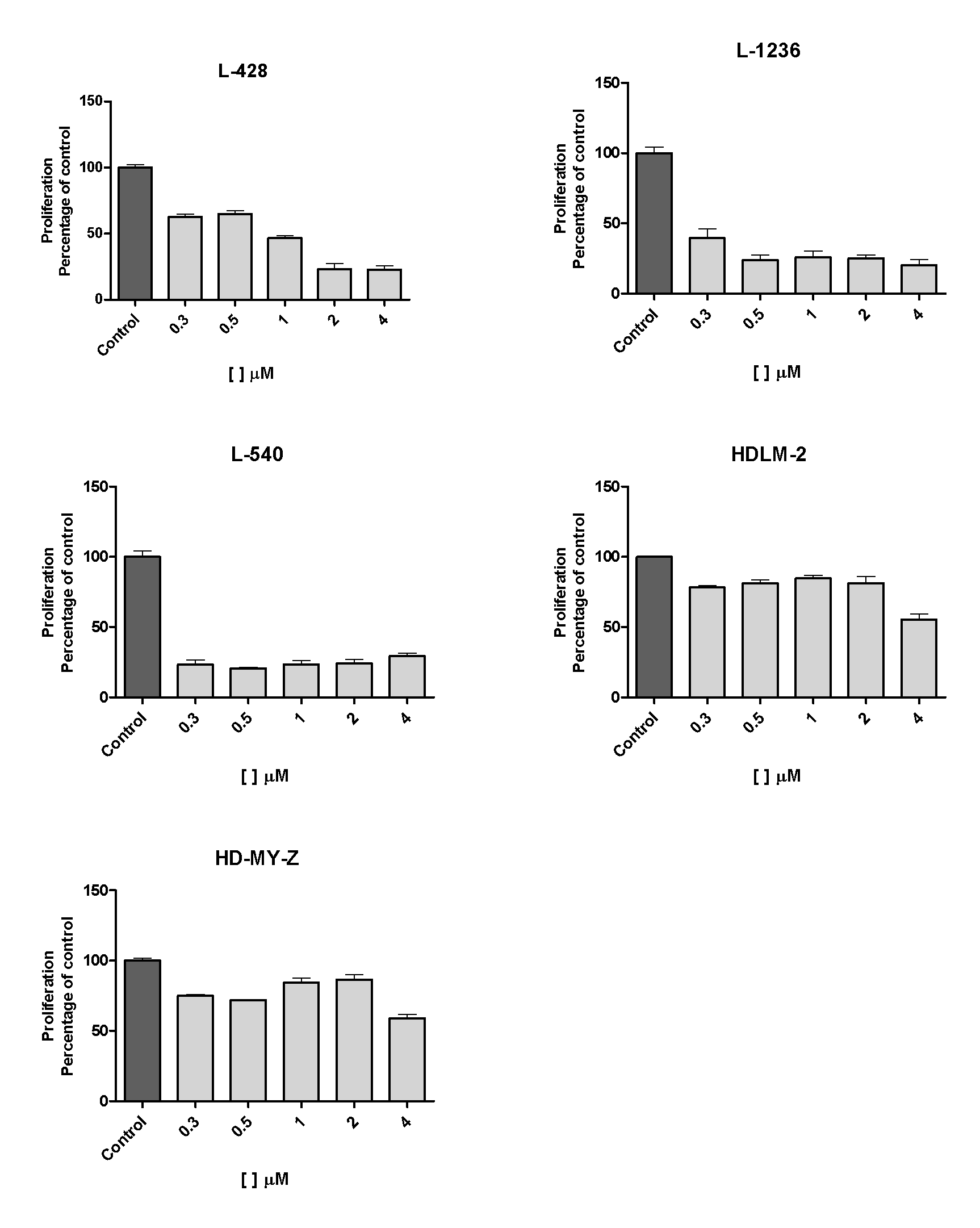

Supplement: Figure S2 — Proliferation analysis after 48 h of Lestaurtinib treatment at increasing doses up to 4 µM in L-428, L-1236, L-540, HDLM-2 and HD-MY-Z cell lines. The data are shown as mean ± SEM of three independent replicates. (TIF) [file pone.0018856.s002.tif]

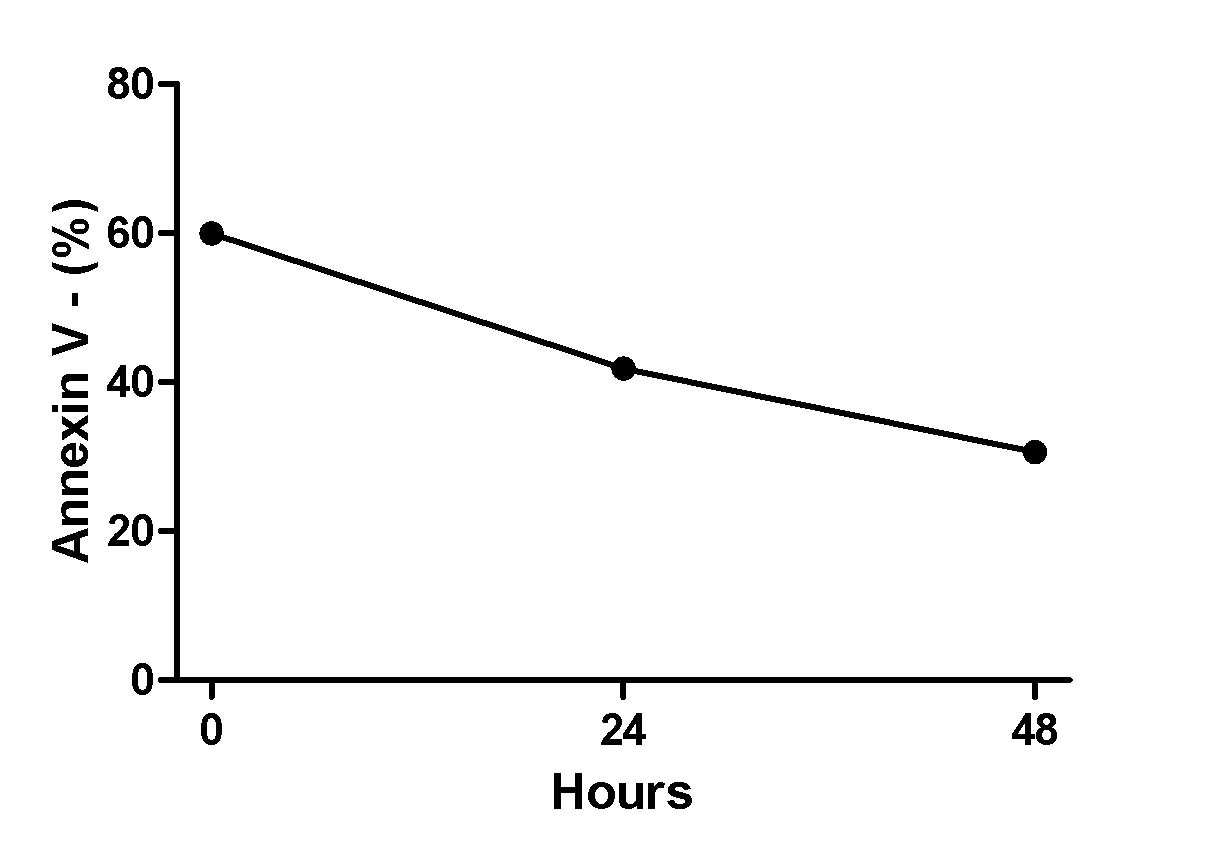

Supplement: Figure S3 — Cell viability analysis (negative Annexin V) of lymph node cells cultured up to 48 h with growth media (RPMI1640 with 10% FBS). (TIF) [file pone.0018856.s003.tif]
